# Palmitoylethanolamide Counteracts Enteric Inflammation and Bowel Motor Dysfunctions in a Mouse Model of Alzheimer’s Disease

**DOI:** 10.3389/fphar.2021.748021

**Published:** 2021-09-29

**Authors:** Vanessa D’Antongiovanni, Carolina Pellegrini, Luca Antonioli, Laura Benvenuti, Clelia Di Salvo, Lorenzo Flori, Rebecca Piccarducci, Simona Daniele, Alma Martelli, Vincenzo Calderone, Claudia Martini, Matteo Fornai

**Affiliations:** ^1^ Department of Clinical and Experimental Medicine, University of Pisa, Pisa, Italy; ^2^ Department of Pharmacy, University of Pisa, Pisa, Italy; ^3^ Interdepartmental Research Center “Nutrafood: Nutraceutica e Alimentazione per la Salute”, University of Pisa, Pisa, Italy; ^4^ Interdepartmental Research Center “Biology and Pathology of Ageing”, University of Pisa, Pisa, Italy

**Keywords:** alzheimer’s disease, colonic dysmotility, enteric glial cells, enteric gliosis, intestinal inflammation, palmitoylethanolamide, SAMP8 mice, toll-like receptor

## Abstract

Palmitoylethanolamide (PEA), an endogenous lipid mediator, is emerging as a promising pharmacological agent in multiple neurodegenerative disorders for its anti-inflammatory and neuroprotective properties. However, its effects on enteric inflammation and colonic dysmotility associated with Alzheimer’s disease (AD) are lacking. This study was designed to investigate the beneficial effect of PEA administration in counteracting the enteric inflammation and relieving the bowel motor dysfunctions in an AD mouse model, SAMP8 mice. In addition, the ability of PEA in modulating the activation of enteric glial cells (EGCs), pivotally involved in the pathophysiology of bowel dysfunctions associated with inflammatory conditions, has also been examined. SAMP8 mice at 4 months of age were treated orally with PEA (5 mg/kg/day) for 2 months. SAMR1 animals were employed as controls. At the end of treatment, parameters dealing with colonic motility, inflammation, barrier integrity and AD protein accumulation were evaluated. The effect of PEA on EGCs was tested in cultured cells treated with lipopolysaccharide (LPS) plus β-amyloid 1–42 (Aβ). SAMP8 treated with PEA displayed: 1) an improvement of *in vitro* colonic motor activity, citrate synthase activity and intestinal epithelial barrier integrity and 2) a decrease in colonic Aβ and α-synuclein (α-syn) accumulation, S100-β expression as well as enteric IL-1β and circulating LPS levels, as compared with untreated SAMP8 mice. In EGCs, treatment with PEA counteracted the increment of S100-β, TLR-4, NF-κB p65 and IL-1β release induced by LPS and Aβ. These results suggest that PEA, under a condition of cognitive decline, prevents the enteric glial hyperactivation, reduces AD protein accumulation and counteracts the onset and progression of colonic inflammatory condition, as well as relieves intestinal motor dysfunctions and improves the intestinal epithelial barrier integrity. Therefore, PEA represents a viable approach for the management of the enteric inflammation and motor contractile abnormalities associated with AD.

## Introduction

Mild cognitive impairment identifies a clinical condition between age-related cognitive decline and dementia and represents a prodromal stage before the development of Alzheimer’s disease (AD) (Murman, 2015). AD is one of the most common neurodegenerative disorders, characterised by a progressive memory decline, cognitive impairment, amyloid β1-42 (Aβ) plaque accumulation, neurofibrillary tangle of hyperphosphorylated tau (p-tau) protein and occurrence of neurogenic/inflammatory responses in the central nervous system (Scheltens et al., 2016). In addition, AD patients are often characterized by functional digestive disturbances, including infrequent bowel movements, constipation, and defecatory disorder (D’Antongiovanni et al., 2020b; Pellegrini et al., 2020).

In the last years, it has been proposed that alterations of enteric bacteria-neuro-immune network may contribute to the onset of bowel motor disturbances associated with AD (Pellegrini et al., 2018a; Mancuso and Santangelo, 2018). In this regard, pre-clinical and human studies have reported that AD is associated with changes in gut microbiota composition, colonic accumulation of Aβ and p-tau tangle-like structures as well as signs of enteric inflammation, which could lead to enteric motor dysfunctions (Joachim et al., 1989; Puig et al., 2015; Piccarducci et al., 2019; Pellegrini et al., 2020). In this respect, interesting evidence obtained from studies on the Senescence-Accelerated Mouse-prone 8 (SAMP8) mouse model indicate that, in the early stages of AD, changes in gut microbiota composition and impairment of intestinal epithelial barrier (IEB) permeability can promote enteric AD protein accumulation, which, in turn, can shape enteric neurogenic/inflammatory responses, thus contributing to gut dysfunctions (Pellegrini et al., 2020). In line with this view, other studies performed on SAMP8 and AβPP/PS1 transgenic AD mouse models observed the accumulation of intestinal Aβ and amyloid precursor protein, enteric inflammation, mitochondrial dysfunction along with enteric glial activation and gut dysbiosis in the early stages of AD before the full development of brain pathology (Joachim et al., 1989; Puig et al., 2015; Piccarducci et al., 2019; Pellegrini et al., 2020). Bowel motor disturbances in AD patients contribute significantly to AD morbidity and complicate their clinical management (Puig et al., 2015; Doi et al., 2019; Camilleri, 2021). In this regard, no specific treatments are currently available to manage gut alterations occurring in such patients and, therefore, the identification of novel pharmacological entities able to prevent or alleviate gut dysfunctions associated with AD represents an area of interest to the scientific community.

Recently, palmitoylethanolamide (PEA), an endogenous lipid mediator, is emerging as a promising pharmacological agent in multiple neurodegenerative disorders for its anti-inflammatory and neuroprotective properties (Scuderi et al., 2012, 2014; Beggiato et al., 2019, 2020). However, the properties of this compound in counteracting the intestinal dysfunctions associated with AD are largely unknown. Currently, some studies provided evidence about an anti-inflammatory effect of PEA in blunting the intestinal inflammation in a murine model of 2,4-dinitrobenzene sulfonic acid (DNBS)-induced colitis and accelerated transit induced by administration of oil of mustard as well as in counteracting the intestinal injury due to ischaemia reperfusion (Capasso et al., 2001, 2014; Di Paola et al., 2012; Borrelli et al., 2015). In support of these findings, Esposito et al. (2014) provided evidences about the putative efficacy of PEA in counteracting intestinal inflammation and dysmotility in mice with dextran sulphate sodium (DSS)-induced colitis and patients with ulcerative colitis (UC) (Esposito et al., 2014). In particular, the authors observed beneficial effect of PEA in counteracting motor dysfunctions and enteric inflammatory processes, through the modulation of enteric glia cells (EGCs) (Esposito et al., 2014), leading to hypothesize a potential application of PEA as a suitable tool for the management of GI dysfunctions associated with AD.

Based on these premises, the present study was designed to investigate the beneficial effect of PEA administration in counteracting the enteric inflammation and relieving the bowel motor dysfunctions in an AD mouse model before the full development of brain pathology. In addition, the ability of PEA in modulating the enteric glial activation has also been examined.

## Materials and Methods

### Experiments on Animals

#### Animals

SAMP8 mice (4 months old), a spontaneous genetic model of AD, and their control strain, Senescence-Accelerated Mouse-Resistant 1 (SAMR1), were purchased from ENVIGO Srl (San Pietro al Natisone UD, Italy). The animals were fed with regular laboratory chow and had free access to tap water and were not utilized for at least 1 week after arriving at the facility. They were held in temperature-controlled rooms, one in a cage, on a 12-h light cycle at 22–24°C and 50–60% humidity.

Their care and handling were following the terms of European Community Council Directive 210/63/UE, which the Italian Government recognized and adopted. The study was approved by the University of Pisa’s Ethical Committee for Animal Experimentation and the Italian Ministry of Health (Authorization No. 875/2018-PR).

The SAMP8 mouse is an accelerated senescence strain that exhibits spontaneously early learning and memory deficits (Butterfield and Poon, 2005; Canudas et al., 2005). Notably, this model exhibits the same clinical and pathophysiological features of AD patients, including Aβ proteins in hippocampal granules, p-tau protein, a decline in choline acetyltransferase activity along with an increase in α-synuclein (α-syn), oxidative damage, presenilin, neuronal nitric oxide synthase and glutamate levels (Butterfield and Poon, 2005). In addition, SAMP8 mice starting from 6 months of age shows digestive functional disturbances (Pellegrini et al., 2020), thus representing a valuable model to investigate the beneficial properties of novel drugs on colonic inflammatory and motor contractile abnormalities associated with AD.

### Experimental Design

**FIGURE 1:**
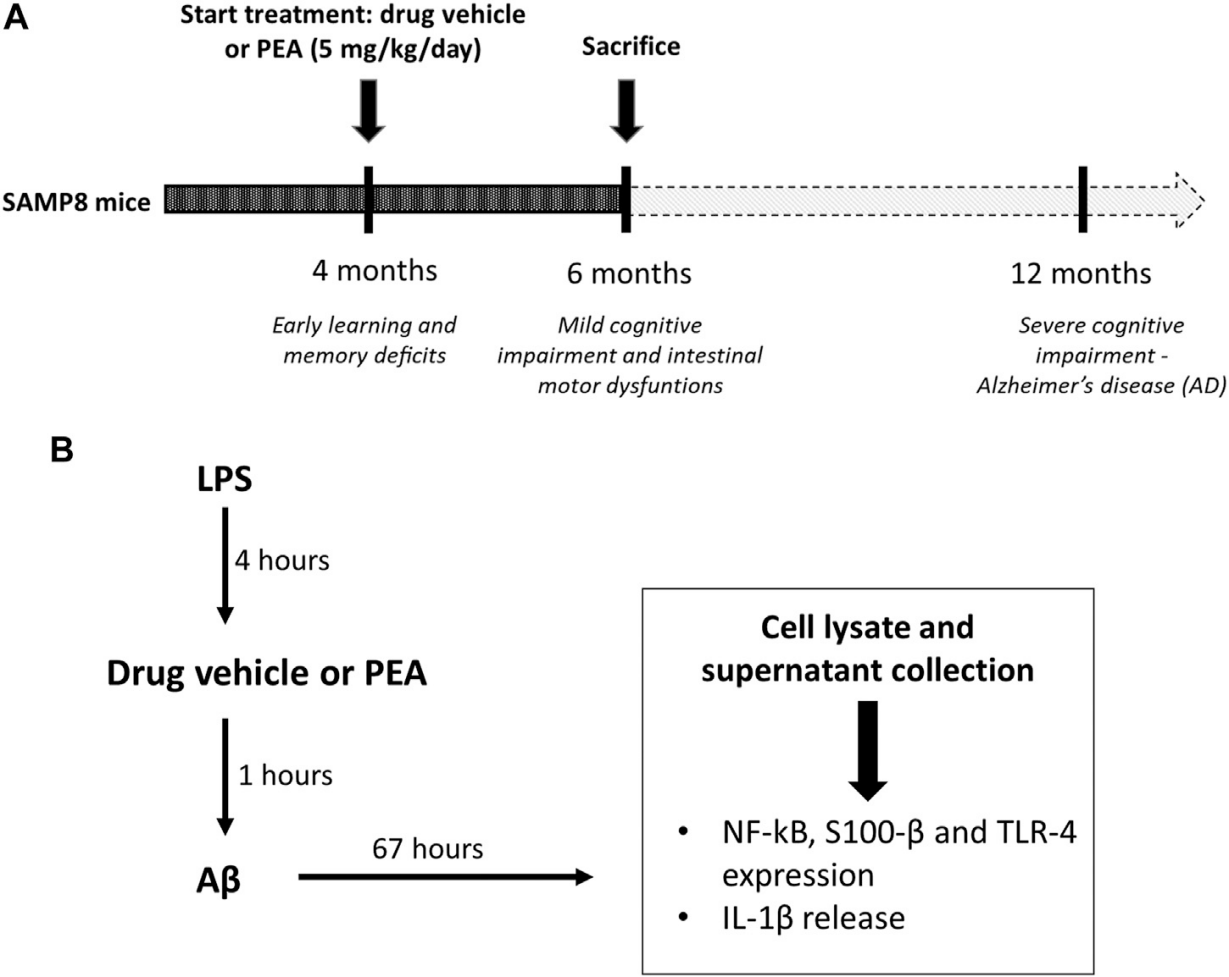
Schematic representation of **(A)** *in vivo* treatment of SAMP8 mice with drug vehicle or PEA (5 mg/kg/day) for 2 months and **(B)** the design of experiments on cultured EGCs: EGCs were treated for 4 h with LPS (1 μg/ml). Then, cells were incubated for 1 h with PEA or drug vehicle before the addition of Aβ (1 μM, 67 h). On the third day, cells were lysed for analysis of S100-β, NF-κB p65 and TLR-4 expression and the culture media were collected for analysis of IL-1β release. *Abbreviations*: Aβ, amyloid β1-42; EGC, enteric glial cell; LPS, lipopolysaccharide; NF-κB p65, nuclear factor-κB p65; PEA, palmitoylethanolamide; S100-β, S100 Calcium Binding Protein B; TLR-4, Toll-like Receptor-4.

Based on previous evidence (Piccarducci et al., 2019; Pellegrini et al., 2020) showing an impairment of cognitive and intestinal motor dysfunctions in SAMP8 mice starting from 6 months of age, the attention was focused on SAMP8 animals at 6 months of age, in order to examine the putative beneficial effects of PEA administration on the intestinal inflammation and bowel motor dysfunctions since the early stages of AD. SAMP8 and SAMR1 animals at 4 months of age were treated orally with PEA (5 mg/kg/day) for 2 months. Subgroups of animals received the drug vehicle and served as controls (Figure 1A). The dose of PEA was selected on the basis of previous studies (Vaia et al., 2016; Facchinetti et al., 2020; Petrosino and Moriello, 2020). In addition, preliminary experiments were performed to assay increasing doses of PEA (1, 5 and 10 mg/kg) on the intestinal inflammation and bowel motor dysfunctions in SAMP8 mice (data not shown). Effective dose of PEA (5 mg/kg) was then selected because suitable for better appreciating the effects of test drug on colonic contractile activity, inflammation, and enteric glial activation.

At the end of treatments, animals were euthanized and tissues were processed for functional experiments and other assays, as described below.

### Recording of Colonic Contractile Activity

The contractile activity of colonic muscle preparations was recorded as previously described (Antonioli et al., 2006, 2011; Pellegrini et al., 2021). Following sacrifice, the abdomen was promptly opened, and the colon was removed and put in Krebs solution at 37°C. Colon specimens were opened along the mesenteric insertion and cut into strips of approximately 3 mm in width and 10 mm in length. The colonic specimens were set up in organ baths containing Krebs solution at 37°C, bubbled with 95% O_2_ + 5% CO_2_. Krebs solution had the following composition: NaCl 113 mM, NaHCO_3_ 25 mM, KCl 4.7 mM, CaCl2 2.5 mM, MgSO_4_ 1.2 mM, KH_2_PO_4_ 1.2 mM and glucose 11.5 mM (pH 7.4 ± 0.1). The preparations were connected to isometric force transducers (constant load = 0.5 g) and the mechanical activity was recorded by BIOPAC MP150 (2Biological Instruments, Besozzo, Italy). A BM-ST6 stimulator (Biomedica Mangoni, Pisa, Italy) was used to provide electrical stimulation through a pair of coaxial platinum electrodes, located 10 mm from the longitudinal axis of each preparation. Preparations were equilibrated for at least 30 min and challenged with electrical stimulation (ES; 10-s single trains of square wave pulses, 0.5 ms, 30 mA), and the tests began when reproducible responses were obtained (on average after two or three stimulations). Each preparation’s tension was normalized by wet tissue weight and expressed as grams per Gram of wet tissue (g/g tissue).

The appropriate ES frequency, as well as concentrations of exogenous carbachol and substance P (SP) were selected in accordance with previous experiments (Pellegrini et al., 2016).

### Design of Functional Experiments

In the first set of experiments, ES-induced contractions were recorded from colonic preparations maintained in standard Krebs solution.

In the second series of experiments, colonic tissues were maintained in Krebs solution containing N-ω-nitro-L-arginine methylester (L-NAME, nitric oxide synthase inhibitor, 100 µM), N-acetyl-l-tryptophan 3,5-bis(trifluoromethyl) benzylester (L-732,138, neurokinin NK_1_ receptor antagonist, 10 μM, guanethidine (adrenergic blocker 10 µM), 5-fluoro-3-[2-[4-methoxy-4-[[(R)-phenylsulphinyl]methyl]-1-piperidinyl]ethyl]-1H-indole (GR159897, NK_2_ receptor antagonist, 1 µM) and (R)-[[(2-phenyl-4-quinolinyl)carbonyl]amino]-methyl ester benzeneacetic acid (SB218795, NK_3_ receptor antagonist, 1 µM), in order to examine the patterns of colonic contractions driven by excitatory nerve cholinergic pathway.

In the third set of experiments, ES-evoked contractions were recorded from colonic preparations maintained in Krebs solution containing L-NAME, guanethidine, atropine sulphate (muscarinic receptor antagonist, 1 µM), GR159897 and SB218795, in order to examine the colonic excitatory motor responses mediated by the tachykininergic NK_1_ receptors pathway.

In the fourth and fifth set of experiments, colonic contractions were evoked by direct pharmacological activation of receptors located on smooth muscle cells. For this purpose, colonic preparations were maintained in Krebs solution containing tetrodotoxin (TTX, 1 µM) and stimulated with carbachol (10 µM) or exogenous SP (1 µM) to assess cholinergic and tachykininergic contractile responses, respectively.

### Quantification of Colonic Neurodegenerative Disorders-Related Protein: Aβ, T-tau, and α-syn

The evaluation of the NDs-related misfolded proteins levels in colonic tissue was assessed by a “home-made” sandwich enzyme-linked immunosorbent assay (ELISA) (Venegas et al., 2017; Nakanishi et al., 2018; Pellegrini et al., 2020). Briefly, an antibody directed against a specific epitope of the interested protein was coated to wells of a 96-wells polystyrene plate diluted in poly-L-ornithine (dissolved in 50 mM NaHCO3 pH 9.6) and it was incubated overnight at 4°C. After washes, the bovine serum albumin (BSA) 1% was added to each well and incubated at 37°C to block non-specific sites. Then, colonic samples were added to wells and incubated at 25°C. Following extensive washes, a primary antibody directed against a different epitope of the interested protein and then a secondary antibody, conjugated with the horseradish peroxidase (HRP) and directed versus the primary antibody, were employed and incubated at 37°C under continuous shaking. Lastly, a chromogenic substrate (3,3′,5,5′-tetramethylbenzidine, TMB) was added and the absorbance was read at 450 nm following the addition of the stop solution (H_2_SO_4_) to block the colorimetric reaction. All the measurements were performed in duplicate to achieve a minimal inter-assay variability. The concentration of the interested protein was calculated by the interpolation of the absorbance values into the standard curve built with the relative recombinant human protein. The employed antibodies for the assay are schematically reported in Table 1.

**TABLE 1:** Antibodies employed in the “home-made” sandwich enzyme-linked immunosorbent assay (ELISA). For each investigated protein, the respective coating, primary, and secondary antibodies employed for the assay are listed. The catalogue number, brand, class type antibody, and immunogen are also reported for all antibodies.

Protein	Coating antibody	Primary antibody	Secondary antibody
Aβ	#44–344, Invitrogen (Waltham, United States)	sc-28365, Santa Cruz (Dallas, United States) Mouse monoclonal antibody (recognizing full length protein)	#31430, ThermoFisher Scientific (Waltham, United States) Goat anti-mouse IgG (HRP)
	Rabbit polyclonal antibody (recognizing aa 36–42, C-terminal)		
t-tau	sc-32274, Santa Cruz (Dallas, United States)	ab109392, abcam (Cambridge, United Kingdom)	A6154, Sigma-Aldrich (St. Louis, MO, United States) Goat anti-rabbit IgG (HRP)
	Mouse monoclonal antibody (recognizing C-terminal)	Rabbit monooclonal antibody (recognizing N-terminal)	
α-syn	NBP2-15365, Novus Biological (Centennial, United States) Rabbit polyclonal antibody (recognizing full length protein)	sc-514908, Santa Cruz (Dallas, United States)	#31430, ThermoFisher Scientific (Waltham, United States) Goat anti-mouse IgG (HRP)
	Mouse monoclonal antibody (recognizing aa 2–24, N-terminal)		

Aβ, amyloid β1-42; α-syn, α-synuclein; t-tau, total tau.

### Evaluation of Citrate Synthase Activity on Colon Samples

The colon samples used for the enzymatic assay were thawed out and homogenized in a cold buffer (Sucrose 250 mM, Tris 5 mM, EGTA 1 mM, Triton X-100 0.02%; pH 7,4) at 4°C using GentleMACS dissociator (Miltenyi Biotec, Bologna, Italy). The homogenate obtained was centrifuged at 12.000 g for 15 min at 4°C (EuroClone, Speed Master 14 R centrifuge, Milan, Italy). The supernatant was removed and stored on ice, the pellet was discarded. The protein assay was performed on the supernatant by Bradford assay for total proteins determination. The protein assay was used to obtain the 0.5 mg/ml and then used 1 μg of proteins per well.

The supernatants were diluted in Tris-buffer (100 mM; pH 8,2). 5,5′-dithiobis-2-nitrobenzoic acid (100 μM) and acetyl-coenzyme A (100 μM) were added to each sample. The assay was performed in 96 multi-well plates and the reaction started by the addition of oxaloacetic acid (500 μM). The reaction was followed spectrophotometrically at 37°C every 30 s for 15 min at the wavelength of 412 nm (EnSpire, PerkinElmer, Waltham, MA, United States). Linear regression was calculated with different concentrations of citrate synthase (Sigma-Aldrich, St. Louis, MO, United States). Citrate synthase activity was expressed in mU/mL.

### Western Blot Analysis

The colon was collected from mice and flushed of fecal content with ice-cold phosphate-buffered saline (PBS), as described previously (Antonioli et al., 2021). Tissues were minced and homogenized using a Potter-Elvehjem Grinder homogenizer on ice in 20% (w/v) TNE lysis buffer (50 mM Tris-HCl pH 7.4, 100 mM NaCl, 0.1 mM EDTA, 1% NP-40, 1% SDS, 0.1% DOC) with proteases and phosphatases inhibitors. Samples were then sonicated and boiled for 5 min at 95°C. Proteins were quantified with the Bradford assay. Total lysates were run on a 4–20% Criterion™ TGX™ Precast Midi Protein Gel (Bio-Rad, Hercules, CA, United States) and then transferred to PVDF membranes (Trans-Blot Turbo^TM^ PVDF Transfer packs, Biorad, Hercules, CA, United States). Membranes were blocked with 3% BSA diluted in Tris-buffered saline (TBS; 20 mM Tris-HCl, pH 7.5, 150 mM NaCl) with 0.1% Tween 20. Primary antibodies against β-actin (ab8227, Abcam, Cambridge, United Kingdom), claudin-1 (sc-166338, Santa Cruz, Dallas, United States), occludin (ab167161, Abcam, Cambridge, United Kingdom), S100-β (ab52642, Abcam, Cambridge, United Kingdom), TLR-4 (ab22048, Abcam, Cambridge, United Kingdom) and ZO-1 (ab96587, Abcam, Cambridge, United Kingdom) were used. Secondary antibodies were obtained from Abcam (anti-mouse ab97040 and anti-rabbit ab6721). Protein bands were detected with ECL reagents (Clarity Western ECL Blotting Substrate, Biorad, Hercules, CA, United States). Densitometry was performed by IBright Analysis software.

### Evaluation of Tissue IL-1β Levels

Tissue interleukin (IL)-1β levels were quantified, as previously described (Antonioli et al., 2020), using a commercial ELISA Kit (Abcam, Cambridge, United Kingdom). Briefly, colon tissues, previously collected and stored at −80°C, were thawed, weighed, and homogenized in PBS (0,4 ml/20 mg of tissue) at 4°C, and centrifuged for 5 min at 10.000 g. Aliquots of 100 µL were used to perform the assay. IL-1β levels were expressed as picograms per milligram (pg/mg) of protein.

### Evaluation of Plasma LBP

Plasma lipopolysaccharide-binding protein (LBP) levels were quantified using a commercial ELISA Kit (Abcam, Cambridge, United Kingdom). For the assay, aliquots (100 µL) of plasma were used. LBP concentrations were expressed as nanograms per milliliter (ng/ml).

### Drugs and Reagents

Aβ, atropine sulphate, carbachol, Dulbecco’s modified Eagle’s medium (DMEM), fetal bovine serum (FBS), guanethidine monosulphate, lipopolysaccharide (LPS), PEA and PBS were purchased from Sigma Aldrich (St. Louis, MO, United States). L-NAME, L-732,138, GR159897, SB218795, SP and TTX were purchased from Tocris (Bristol, United Kingdom).

### Experiments on Cultured Enteric Glial Cells

#### Cell Culture

Rat-transformed enteric glial cells (EGCs) were acquired from ATCC^®^ (EGC/PK060399egfr; ATCC®CRL-2690.; Manassas, VA, United States). Cells were grown and maintained in DMEM supplemented with 100 unit/mL penicillin-streptomycin, 10% FBS and 2 mM glutamine in a humidified atmosphere of 5% CO_2_ at 37°C.

#### Stimulation Protocol

EGCs were seeded at a density of 1 × 10^6^ cells in 6-well plates containing culture medium. To mimic the *in vivo* features of AD, cells were treated with LPS (1 μg/ml, 4 h) before treatment with Aβ (1 µM), in the presence or in the absence of 0.1 µM PEA. Controls were run in parallel. The concentrations of Aβ, LPS, and PEA were selected in accordance with previous studies (Scuderi et al., 2012; Antonioli et al., 2020; Beggiato et al., 2020). The details of all treatments are shown in Figure 1B.

#### Western Blot Analysis

Cells were lysed as previously described (Fazzini et al., 2014; Smoktunowicz et al., 2016). Proteins were quantified with the Bradford assay. Proteins were separated onto a pre-cast 4–20% polyacrylamide gel (Mini-PROTEAN® TGX gel, Biorad, Hercules, CA, United States) and transferred to PVDF membranes (Trans-Blot® Turbo^TM^ PVDF Transfer packs, Biorad, Hercules, CA, United States). Membranes were blocked with 3% BSA diluted in Tris-buffered saline (TBS; 20 mM Tris-HCl, pH 7.5, 150 mM NaCl) with 0.1% Tween 20. Primary antibodies against β-actin (ab8227, Abcam, Cambridge, United Kingdom), nuclear factor-κB p65 (NF-κB p65, sc-8008, Santa Cruz, Dallas, United States) S100-β (ab52642, Abcam, Cambridge, United Kingdom) and TLR-4 (ab22048, Abcam, Cambridge, United Kingdom) were used. Secondary antibodies were obtained from Abcam (anti-mouse ab97040 and anti-rabbit ab6721). Protein bands were detected with ECL reagents (Clarity Western ECL Blotting Substrate, Biorad, Hercules, CA, United States). Densitometry was performed by IBright Analysis software.

#### Assessment of IL-1β Release From EGCs

The release of IL-1β into culture medium was measured by ELISA kit (Abcam, Cambridge, United Kingdom), following the manufacturer’s protocol. After cell stimulation, the medium was collected and centrifuged at 800 rpm for 5 min to obtain cell-free supernatants. Supernatants (150 μL) were then used for the assay.

### Statistical Analysis

Data are presented as mean ± SEM and analyzed by GraphPad Prism 7.0 (GraphPad Software Inc., San Diego, CA, United States). Statistical significances were determined by one-way ANOVA followed by Tukey’s post hoc test. Statistical analysis for citrate synthase activity was performed with Student’s t-test. A p value < 0.05 was considered significantly different.

## Results

The administration of PEA to SAMR1 mice did not elicit any significant change in both *in vitro* colonic contractile activity and enteric inflammatory parameters, as well as in enteric glial activation (data not shown). Therefore, SAMR1 mice treated with drug vehicle were adopted as control group for all the evaluations on the drug under investigation.

### *In Vitro* Colonic Contractile Activity

**FIGURE 2:**
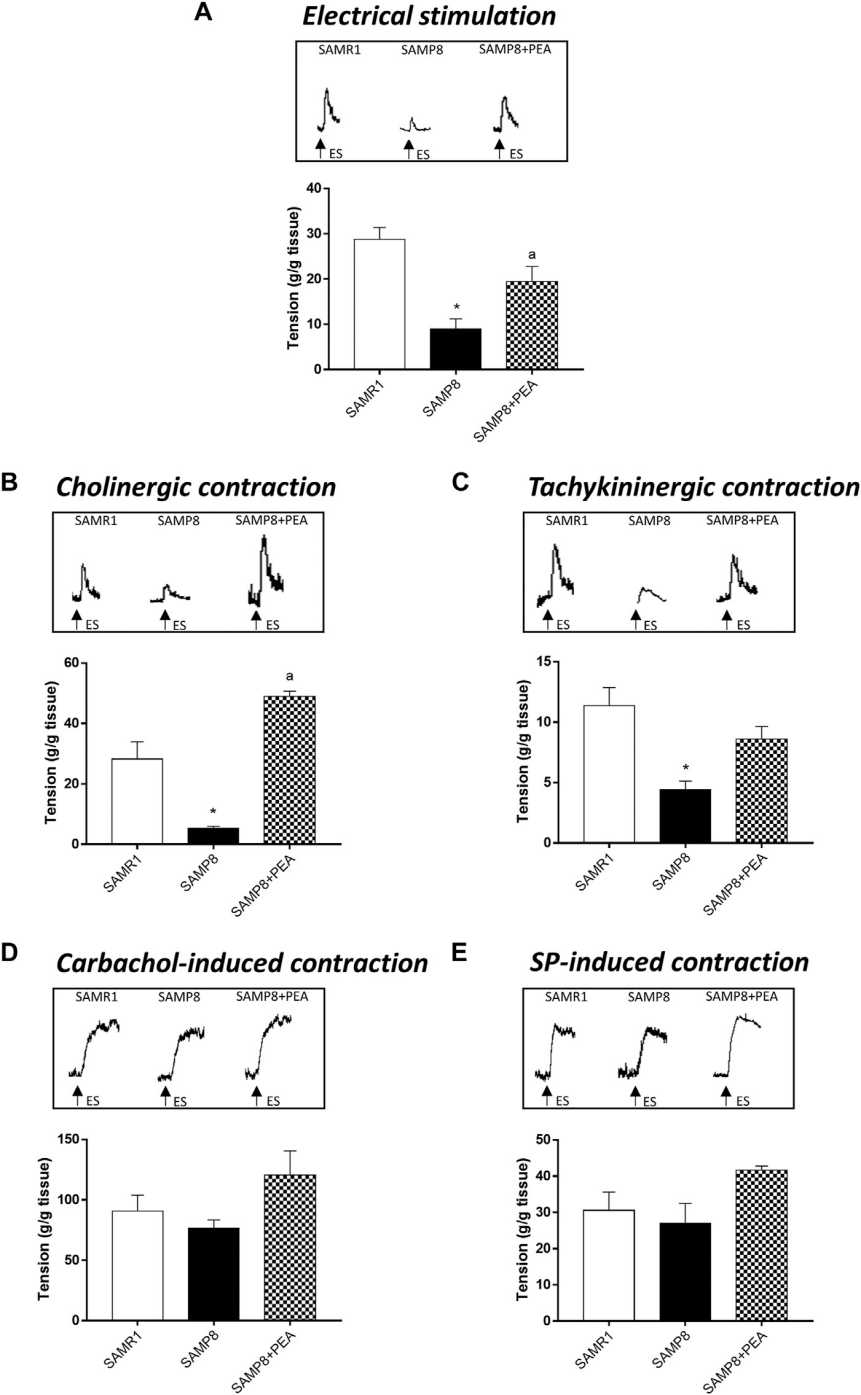
PEA improves the bowel motor dysfunctions associated with AD. Effect of PEA on *in vitro* colonic contractile responses. **(A)** ES (10 Hz), **(B)** cholinergic contractions and **(C)** NK_1_-mediated tachykininergic contractions on contractile activity of colonic longitudinal smooth muscle preparations isolated from SAMR1, SAMP8 and SAMP8 treated with PEA. Contractions evoked by **(D)** carbachol (10 µM) or **(E)** exogenous SP (1 µM) in colonic preparations isolated from SAMR1, SAMP8 and SAMP8 treated with PEA. **(A**–**E)** Tracings in the inset on the top of panels display the contractile responses. Each column represents the mean ± S.E.M. from four animals. One-way ANOVA followed by Tukey post hoc test results: *p < 0.05, significant difference vs. SAMR1 and ^a^ *p* < 0.05, significant difference vs. SAMP8; *Abbreviations*: ES, electrical stimulation; PEA, palmitoylethanolamide; SP, substance P.

In colonic longitudinal muscle preparations maintained in standard Krebs solution, the contractions evoked by ES accounted for 28.69 ± 2.67 g/g tissue for SAMR1 mice (Figure 2A). In colonic preparations from SAMP8 mice, electrically evoked contractions were significantly reduced (8.98 ± 2.25 g/g tissue) (Figure 2A). Treatment with PEA significantly counteracted the reduction of electrically evoked contractions in SAMP8 mice (19.46 ± 3.30 g/g tissue) (Figure 2A).

In colonic preparations maintained in Krebs solution added with L-NAME, guanethidine, L-732,138, GR159897 and SB218795, the electrically evoked atropine-sensitive cholinergic contractions were significantly reduced in the SAMP8 mice, as compared with SAMR1 (5.32 ± 0.65 and 28.21 ± 5.74 g/g tissue, respectively) (Figure 2B). In this setting, PEA significantly improved the electrically evoked cholinergic contractions in SAMP8 mice, thus suggesting an improvement of enteric cholinergic neuromuscular pathway (Figure 2B).

In colonic preparations maintained in Krebs solution containing L-NAME, guanethidine, atropine, GR159897 and SB218795, the ES-induced NK_1_-mediated contractions were significantly reduced in SAMP8 mice, as compared with SAMR1 (11.35 ± 1.52 and 4.43 ± 0.69 g/g tissue, respectively) (Figure 2C). Treatment with PEA counteracted, although not significantly, the electrically NK_1_-mediated contractions in SAMP8 mice (Figure 2C).

The stimulation by carbachol or exogenous SP of colonic preparations from SAMR1, SAMP8 and SAMP8 treated with PEA elicited contractions of similar magnitude (90.64 ± 13.38; 76.46 ± 6.89 and 117.9 ± 14.4 g/g tissue, respectively, for carbachol-induced stimulation; 30.53 ± 5.06; 27 ± 5.47 and 40.5 ± 1.40 g/g tissue, respectively, for SP-induced contraction) (Figures 2D,E).

### Colonic NDs-Related Proteins

**FIGURE 3:**
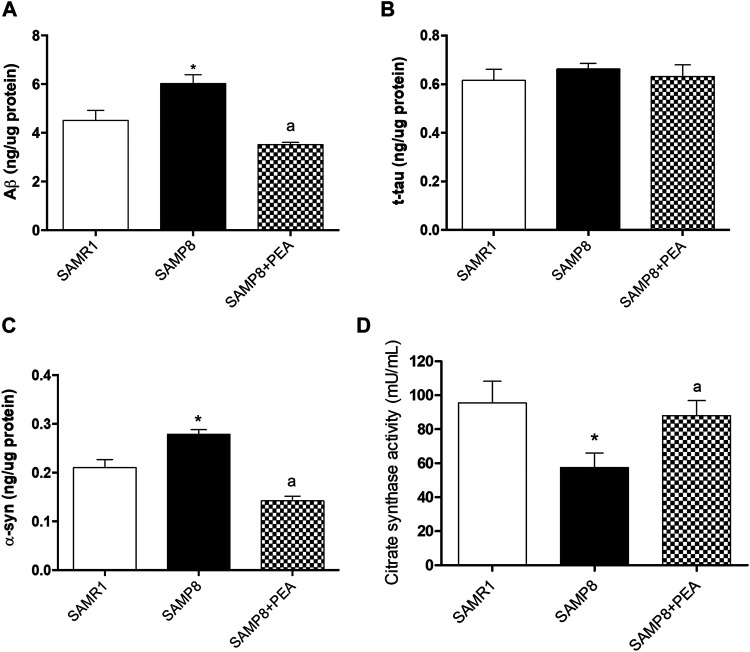
PEA counteracts the accumulation of NDs-related proteins and restores the citrate synthase activity in colonic tissues from SAMR1, SAMP8 and SAMP8 treated with PEA. Quantification of NDs-related proteins: **(A)** Aβ, **(B)** t-tau, and **(C)** α-syn concentrations were assessed by a “home-made” ELISA assay. Each column represents the mean ± SEM. from three animals. **(D)** citrate synthase activity in colonic tissues from SAMR1, SAMP8 and SAMP8 treated with PEA animals. Each column represents the mean ± SEM. from four animals. One-way ANOVA followed by Tukey post hoc test results: *p < 0.05, significant difference vs. SAMR1 and ^a^ *p* < 0.05, significant difference vs. SAMP8.; *Abbreviations*: Aβ, amyloid β1-42; α-syn, α-synuclein; ELISA, enzyme-linked immunosorbent assay; NDs, neurodegenerative disorders; PEA, palmitoylethanolamide; t-tau, total tau.

In order to explore PEA effects in the accumulation of misfolded proteins related to AD pathology, the relative concentrations of NDs-related proteins were assessed in the colon of SAMP8 mice treated with PEA and compared to SAMP8 mice and SAMR1 mice (Figure 3). The concentrations of colonic Aβ were significantly increased in SAMP8 mice compared to SAMR1 mice, confirming already published data (Pellegrini et al., 2020) (Figure 3A). SAMP8 treated with PEA showed significantly decreased concentrations of colonic Aβ compared to untreated SAMP8 mice. Of note, following treatment with PEA the accumulation of colonic Aβ was comparable to that elicited by SAMR1 (Figure 3A), thus denoting a great effect in reducing Aβ levels.

The colonic t-tau levels in SAMP8 mice were comparable to SAMR1 mice, in accordance with previous data (Pellegrini et al., 2020). Furthermore, t-tau concentrations in SAMP8 mice treated with PEA were comparable to untreated SAMP8 and SAMR1 mice (Figure 3B).

The α-syn levels in colon of SAMP8 mice were significantly higher than in SAMR1, thus confirming the previously demonstrated trend of α-syn accumulation in the AD animal model (Pellegrini et al., 2020). Interestingly, the concentration of colonic α-syn was significantly reduced in SAMP8 treated with PEA compared to SAMP8 and even compared to SAMR1 mice (Figure 3C).

### Citrate Synthase Activity in Colonic Tissue

As reported in a previous work (Pellegrini et al., 2020), citrate synthase activity levels recorded in colonic tissues from SAMP8 are significantly reduced compared to those exhibited by SAMR1 mice (57.44 ± 8.51 mU/mL vs 95.44 ± 12.81 mU/mL) (Figure 3D). The administration of PEA to SAMP8 mice significantly prevents the decrease of citrate synthase activity (88.00 ± 0.81 mU/mL) associated with the early ageing of this animal model, assuring the preservation of the mitochondrial functionality (Figure 3D).

### Expression of Toll-like Receptor-4 in Colonic Tissues

**FIGURE 4:**
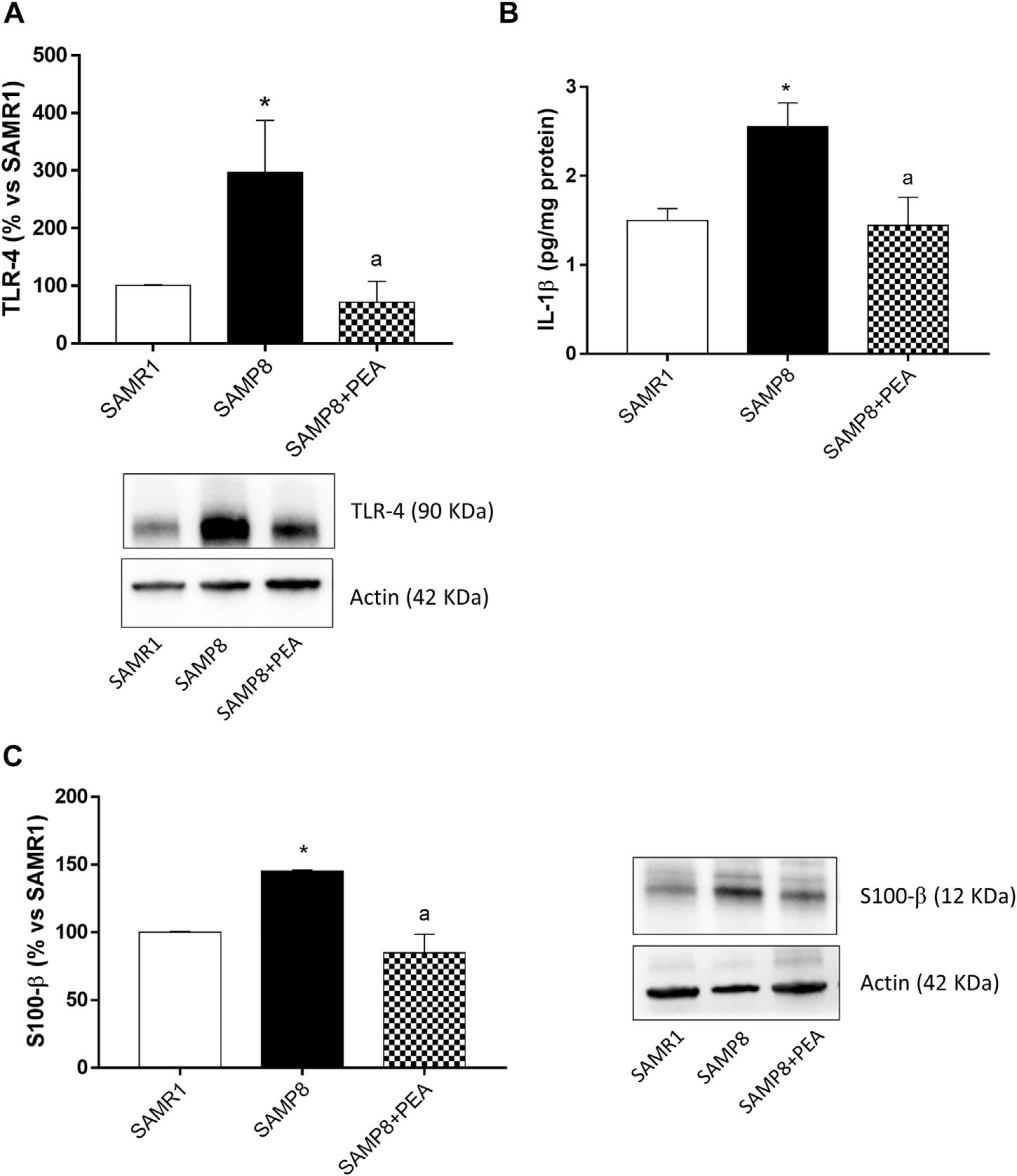
PEA counteracts the intestinal inflammation and enteric gliotic process associated with cognitive decline. **(A)** Representative blots and densitometric analysis of the expression of TLR-4 in colonic tissues from SAMR1, SAMP8 and SAMP8 treated with PEA; **(B)** IL-1β levels in colonic tissues from SAMR1, SAMP8 and SAMP8 treated with PEA and **(C)** representative blots and densitometric analysis of glial marker, S100-β, in colonic tissues from SAMR1, SAMP8 and SAMP8 treated with PEA. Each column represents the mean ± SEM from four animals. One-way ANOVA followed by Tukey post hoc test results: *p < 0.05, significant difference vs. SAMR1 and ^a^ *p* < 0.05, significant difference vs. SAMP8.; *Abbreviations*: IL-1β, interleukin-1β; PEA, palmitoylethanolamide; S100-β, S100 Calcium Binding Protein B; SP, substance P; TLR-4, Toll-like Receptor 4.

The expression of TLR-4, pivotally involved in the occurrence of inflammatory responses, was examined in colonic tissues from SAMR1, SAMP8 and SAMP8 treated with PEA. SAMP8 mice displayed a significant increase in colonic TLR-4 levels, as compared with SAMR1 mice (Figure 4A). Treatment of SAMP8 mice with PEA was associated with a significant decrease in TLR-4 expression levels, as compared with untreated SAMP8 animals (Figure 4A).

### Interleukin-1β Levels in Colonic Tissues

Colonic tissues from SAMP8 mice were characterized by a significant increase in IL-1β, as compared with SAMR1 mice (Figure 4B). Treatment with PEA determined a significant reduction of IL-1β levels in SAMP8 mice, as compared to the levels found in untreated mice (Figure 4B).

### Evaluation of Enteric Glial Activation in SAMP8 Mice

To investigate the ability of PEA in counteracting the enteroglial activation, the expression of glial marker, S100-β, in colonic tissues from SAMR1, SAMP8 and SAMP8 treated with PEA was evaluated. The expression of S100-β was significantly higher in colonic tissues from SAMP8, as compared with SAMR1 mice (Figure 4C). In these settings, treatment with PEA was associated with a significant reduction in the expression level of glial marker S100-β in SAMP8 mice (Figure 4C).

### Assessment of Intestinal Epithelial Barrier Integrity and Permeability in SAMP8 Mice

**FIGURE 5:**
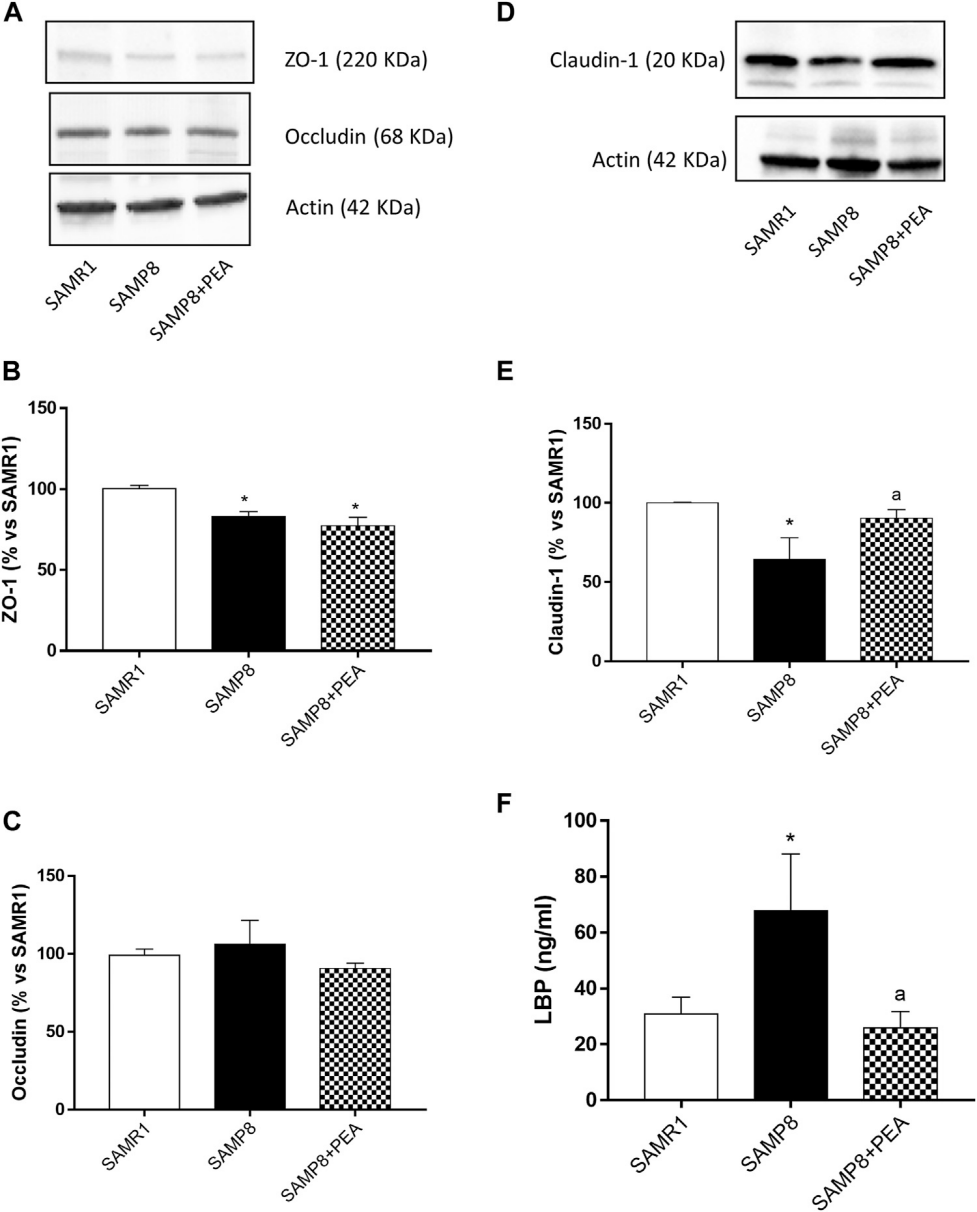
PEA improves the intestinal epithelial barrier integrity in the presence of inflammation. Representative blots and densitometric analysis of the expression of **(A, B)** ZO-1, **(A, C)** occludin and **(D, E)** claudin-1 in colonic tissues from SAMR1, SAMP8 and SAMP8 treated with PEA. **(F)** Circulating LBP in SAMR1, SAMP8 and SAMP8 treated with PEA. Each column represents the mean ± SEM from four animals. One-way ANOVA followed by Tukey post hoc test results: *p < 0.05, significant difference vs. SAMR1 and ^a^ *p* < 0.05, significant difference vs. SAMP8.; *Abbreviations*: LBP, lipopolysaccharide-binding protein; PEA, palmitoylethanolamide; ZO-1, zonulin-1.

SAMP8 mice displayed a significant reduction in the expression levels of ZO-1 and claudin-1, as compared with SAMR1, while no differences were observed in the occludin expression between SAMP8 and SAMR1 mice (Figures 5A–E). Treatment with PEA did not exert significant effects in the expression levels of ZO-1 and occludin in SAMP8 mice, while it significantly increased the claudin-1 expression (Figures 5A–E).

Plasma levels of circulating LPS were significantly higher in SAMP8 than SAMR1 mice (67.8 ± 20.31 ng/ml and 30.93 ± 5.96 ng/ml, respectively) (Figure 5F). PEA administration was associated with a significant decrease in plasma levels of LPS in SAMP8 mice (25.85 ± 5.93 ng/ml) (Figure 5F).

### Effect of PEA in Enteric Glial Activation and Inflammatory Responses in cultured EGCs treated with Aβ and LPS.

Set of *in vitro* experiments were performed to evaluate the effect of PEA in counteracting reactive gliosis and inflammatory responses, as a consequence of enteric glial hyperactivation, in cultured EGCs.

### Expression of S100-β, TLR-4 and NF-κB p65

**FIGURE 6:**
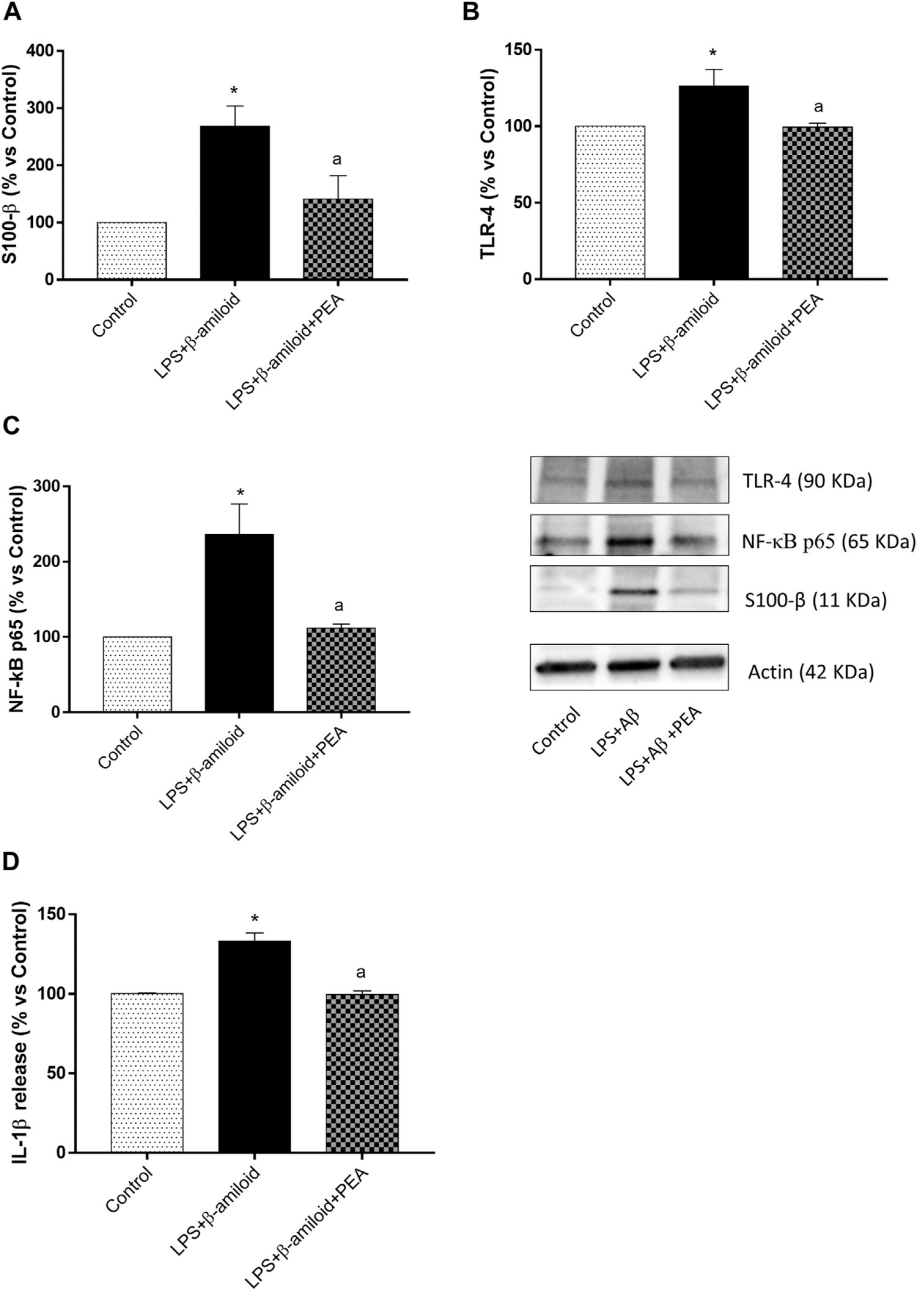
PEA blunts glial pro-inflammatory responses. Representative blots and densitometric analysis of the expression of **(A)** S100-β, **(B)** TLR-4 and **(C)** NF-κB p65 assessed by Western blot assay in cultured EGCs treated with LPS plus Aβ, either alone or in combination with PEA. Each column represents the mean ± SEM (*n* = 4). **(D)** IL-1β levels in the supernatants of EGCs treated with LPS plus Aβ, either alone or in combination with PEA. Each column represents the mean ± SEM (*n* = 4). One-way ANOVA followed by Tukey post hoc test results: *p < 0.05, significant difference vs. SAMR1 and ^a^ *p* < 0.05, significant difference vs. SAMP8.; *Abbreviations*: EGC, enteric glial cell; IL-1β, interleukin-1β; NF-κB p65, nuclear factor-κB p65; PEA, palmitoylethanolamide; S100-β, S100 Calcium Binding Protein B; TLR-4, Toll-like Receptor 4.

EGCs incubated with LPS and Aβ showed a significant increase in S100-β, TLR-4 and NF-κB p65, as compared with control cells (Figures 6A–C). Treatment with 0.1 µM PEA significantly counteracted the increase in the expression of all parameters examined (Figures 6A–C).

### IL-1β Release

Incubation of EGCs with LPS and Aβ induced a significant increase in IL-1β release, as compared with control cells (Figure 6D). Such an increase was counteracted by 0.1 µM PEA (Figure 6D).

## Discussion

Patients with AD often experience digestive functional disturbances, undermining their quality of life and contributing relevantly to morbidity (D’Antongiovanni et al., 2020b; Pellegrini et al., 2020). Several pre-clinical studies allowed to observe that the onset of such bowel motor disturbances could be a consequence of enteric AD protein accumulation, activation of intestinal inflammatory pathways, neuronal loss and enteric glial activation since the earliest stages of brain pathology (Joachim et al., 1989; Puig et al., 2015; Piccarducci et al., 2019; Pellegrini et al., 2020), thus prompting the scientific community to identify novel approaches for the management of motor contractile abnormalities associated with AD.

Currently, the available therapeutic tools to manage AD are mostly focusing on improve cognition and preserve brain functions, leaving out the problem of gut dysmotility. For this reason, the identification of new therapeutic tools for the management of bowel dysfunctions associated with AD represents a significant medical need. In the last years, PEA is emerging as a promising pharmacological agent for its ability to counteract brain neuroinflammation and neurodegeneration in different animal models of AD (Scuderi et al., 2012, 2014; Beggiato et al., 2019, 2020). In addition, pioneering studies have reported beneficial effects of PEA in blunting the acute phase of intestinal inflammation and improving the intestinal motility in murine models of post-inflammatory accelerated transit and UC (Capasso et al., 2001, 2014; Esposito et al., 2014; Borrelli et al., 2015), leading to hypothesize a potential application of PEA in the management of enteric inflammation and intestinal motor dysfunctions associated with AD.

Based on these premises, the aims of the present study were: 1) to investigate the putative effect of PEA in curbing enteric inflammatory processes and improving gut dysmotility in a mouse model of AD and 2) to evaluate, via *in vitro* experiments, the ability of PEA in modulating the activation of EGCs, pivotally involved in the pathophysiology of enteric motor dysfunctions associated with inflammatory conditions (Delvalle et al., 2018; Antonioli et al., 2020; D’Antongiovanni et al., 2020a).

The SAMP8 mouse model at 6 months of age displayed an impairment of excitatory cholinergic and tachykininergic motor contractions, enteric AD protein accumulation and intestinal mitochondrial dysfunctions (a hallmark of Aβ-induced neuronal toxicity in AD) (Pellegrini et al., 2020). In addition, signs of enteric inflammation, which seem to contribute relevantly to the onset of bowel motor dysfunctions (Pellegrini et al., 2018a; 2018b, 2020), were observed in colonic specimens from SAMP8 mice, as documented by the significant increment of TLR-4 expression (receptor subtype widely involved in the occurrence of inflammatory responses) and IL-1β levels. These results are in line with previous pre-clinical and human findings showing the presence of intestinal inflammation in colonic mucosal samples from senescence-accelerated mouse models and AD patients (Piccarducci et al., 2019; Park et al., 2020; Pellegrini et al., 2020).

Treatment with PEA was associated with a normalization of excitatory cholinergic and tachykininergic colonic contractions in SAMP8 mice, thus providing the first experimental demonstration that such pharmacological intervention is able to improve the bowel motor dysfunctions associated with AD. In addition, PEA administration was effective in counteracting the accumulation of AD-related proteins (i.e., Aβ and α-syn) in colonic tissues from SAMP8 mice. This is an interesting point since it is known that the accumulation of AD-related proteins can promote mitochondrial dysfunctions, which, in turn, can trigger the occurrence of enteric neurogenic/inflammatory conditions that could contribute to bowel motor abnormalities (Eckert and Pagani, 2011; Jackson and Theiss, 2020; Pellegrini et al., 2020).

Based on the above considerations, the effect of PEA administration was evaluated on the citrate synthase activity, referred to as a suitable marker of mitochondrial activity, and on colonic inflammation in SAMP8 animals. Treatment with PEA was able to restore the citrate synthase activity and improve tissue inflammatory parameters in SAMP8 mice, thus unraveling, for the first time, a beneficial effect of this compound in counteracting the enteric mitochondrial dysfunction and intestinal inflammation associated with cognitive decline. This finding is in line with previous studies showing a reduction of pro-inflammatory cytokines, such as IL-1β and tumor necrosis factor (TNF), in colonic tissues from UC mice treated with PEA (Esposito et al., 2014; Alhouayek et al., 2015; Borrelli et al., 2015; Hasenoehrl et al., 2017). The mechanism underlying the anti-inflammatory effect of PEA could ascribed, at least in part, to its ability to inhibit the NLRP3 inflammasome/IL-1β pathways as well as to promote macrophage polarization towards the anti-inflammatory M2-type phenotype (Gabrielsson et al., 2017; Rinne et al., 2018; Contarini et al., 2019).

Of interest, a number of investigations have suggested that the persistent condition of enteric inflammation, besides determining intestinal dysfunctions, can also leads to structural and functional changes among the cellular components of the enteric nervous system (ENS), including enteric glia (D’Antongiovanni et al., 2020b). When exposed to inflammation, EGCs acquire a pro-inflammatory phenotype (designated as reactive gliosis) (Ochoa-Cortes et al., 2016), releasing a plethora of inflammatory cytokines [i.e., IL-1β, IL-6 and interferon (INF)-γ], which are thought to take a significant part in the initiation/maintenance of bowel motor dysfunctions (Sharkey, 2015; Delvalle et al., 2018; Antonioli et al., 2020). In this study, SAMP8 mice showed an increase in the expression of S100-β in colonic tissues, suggesting the presence of reactive gliotic processes. PEA administration induced a reduction of enteroglial-derived S100-β protein expression in colonic tissues from SAMP8 mice, indicating that PEA is able to blunt the gliotic reaction. These results are in keeping with previous data showing that PEA administration reduced the expression of S100-β in EGCs derived from murine model of DSS-induced colitis and UC patients (Esposito et al., 2014). Taken together, this evidence allows to hypothesize that the anti-inflammatory effects of PEA are likely to depend, at least in part, on its ability to modulate the EGC activation.

In order to evaluate the effect of PEA in counteracting reactive gliotic processes and inflammatory responses, as a consequence of enteric glial hyperactivation, a set of *in vitro* experiments were performed on cultured EGCs incubated with Aβ (a hallmark of AD) and LPS (to reflect an altered intestinal permeability). Under these conditions, EGCs displayed a hyperactivation, which was blunted by PEA. Despite an inhibitory action of PEA on reactive gliotic processes was reported previously (Esposito et al., 2014), the present study provide evidence, for the first time, of a modulatory action of this compound on glial cells under experimental conditions mimicking AD. This is an interesting point since it is well recognized that the enteric glia holds an active role in the pathogenesis of enteric dysmotility (Capoccia et al., 2015; Sharkey, 2015).

Since reactive glial cells trigger a broad spectrum of alterations, including altered expression and activation of TLRs, activation of pro-inflammatory signaling pathways (i.e., NF-κB p65, considered the main effector of TLR activation) and the release of pro-inflammatory cytokines (Srinivasan and Lahiri, 2015; Ochoa-Cortes et al., 2016), the ability of PEA in counteracting such glial pro-inflammatory responses was examined. In particular, the attention was focused on TLR-4, known to be mainly involved in the detection of bacterial LPS on EGCs (Turco et al., 2014). Stimulation of TLR-4 by LPS or pathogen-associated molecular pattern molecules activates NF-κB p65 signaling with consequent production of several pro-inflammatory cytokines, including IL-1β (Molteni et al., 2016). Likewise, co-treatment of EGCs with LPS and Aβ promoted an increase in TLR-4 and NF-κB p65 expression along with an increase in IL-1β release. Interestingly, such an effect was abrogated when EGCs were incubated with PEA, thus highlighting the ability of this compound in blunting the glial-mediated inflammatory processes. Of note, these results corroborate previous data observed in mice and patients with UC, showing that the anti-inflammatory effects of PEA are mediated by the selective targeting of the S100-β/TLR-4 axis on ECGs, resulting in an inhibition of NF-κB p65 pathway and cytokines release (Esposito et al., 2014).

It has been reported that an abnormal activation of EGCs contributes to the onset and progression of enteric inflammation (Von Boyen and Steinkamp, 2010; Capoccia et al., 2015), which, in turn, besides contributing to bowel motor dysfunctions, could alter the intestinal epithelial barrier (IEB) integrity (Lechuga and Ivanov, 2017; Benvenuti et al., 2020). In particular, it has been observed that IL-1β plays a critical role in the development of IEB dysfunction (Al-Sadi and Ma, 2007; Al-Sadi et al., 2013). In accordance with this evidence, SAMP8 mice were associated with an increased colonic concentration of IL-1β and glial hyperactivation along with an impairment of IEB, as documented by a decreased expression of tight junction proteins and an increment in circulating LPS levels (regarded as an indirect index of intestinal permeability), suggesting an impairment of IEB in concomitance with enteric phlogistic process in early AD animals. Interestingly, treatment with PEA prevented the reduction of ZO-1 and claudin-1 expression as well as the translocation of LPS into the intestinal mucosa in SAMP8 mice, suggesting a protective role of this compound in the maintenance of IEB integrity in the presence of inflammation. In line with these results, a recent paper by Couch et al. (Couch et al., 2019), reported the ability of PEA in preventing the increase of IEB permeability in human intestinal Caco-2 cells exposed to TNF and IFN-γ. In the same study, the authors also observed that the oral consumption of PEA prevented the increase in IEB permeability in the inflamed gut of patients with inflammatory bowel disease (Couch et al., 2019), indicating the use of PEA as an efficacious treatment to counteract the inflammation-induced hyperpermeability.

## Conclusion

The present research work represents a point of extreme novelty suggesting that PEA, under a condition of cognitive decline, can prevent the enteric glial hyperactivation and reduce the accumulation of AD-related proteins as well as counteract the onset and progression of colonic inflammatory condition. In addition, these findings provide evidence, for the first time, that PEA can relieve bowel dysmotility associated with AD, through a normalization of excitatory cholinergic and tachykininergic colonic contractions and improve the IEB integrity. Based on these findings, it is conceivable that PEA, through its ability to counteract the reactive gliotic processes, can blunt effectively the enteric phlogistic processes occurring in the setting of AD with consequent improvement of the bowel motor dysfunctions and the IEB integrity. Therefore, PEA represents a viable approach for the management of the enteric inflammation and motor contractile abnormalities associated with AD.

## Data Availability

The raw data supporting the conclusion of this article will be made available by the authors, without undue reservation.
